# Supplementary material for: T Cell Transcriptional Signatures of Influenza A/H3N2 Antibody Response to High Dose Influenza and Adjuvanted Influenza Vaccine in Older Adults
Source: Viruses. 2022 Dec 11;14(12):2763. doi: 10.3390/v14122763 (PMC9786771; doi:10.3390/v14122763)
Supplement: Supplementary file 1 [file viruses-14-02763-s001.zip › viruses-2015129-supplementary.pdf]

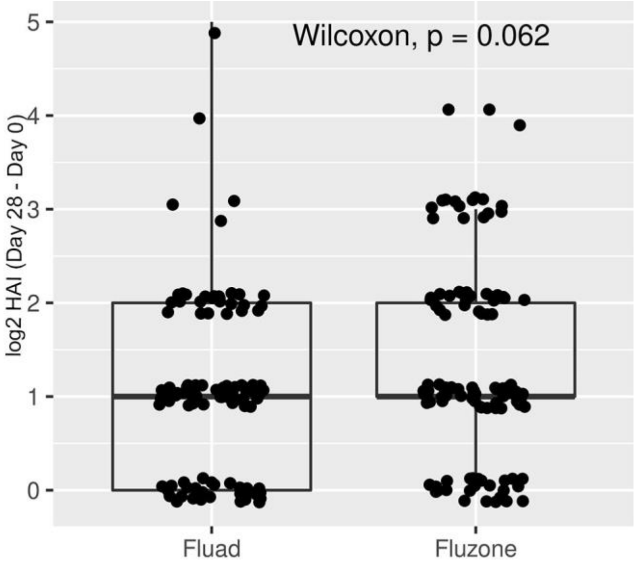

Figure S1. Antibody response by vaccine type. The vertical axis represents the log_2 change in HAI titer from Day 0 to Day 28; a one unit increase in the log2 HAI measure represents a doubling of the HAI titer from Day 0 to Day 28. Each box was plotted using the 25% to 75% interquartile range and the median was represented by the bold line in the box. The "whiskers" extend up to 1.5 times the interquartile range above or below the 75th or 25th percentiles.

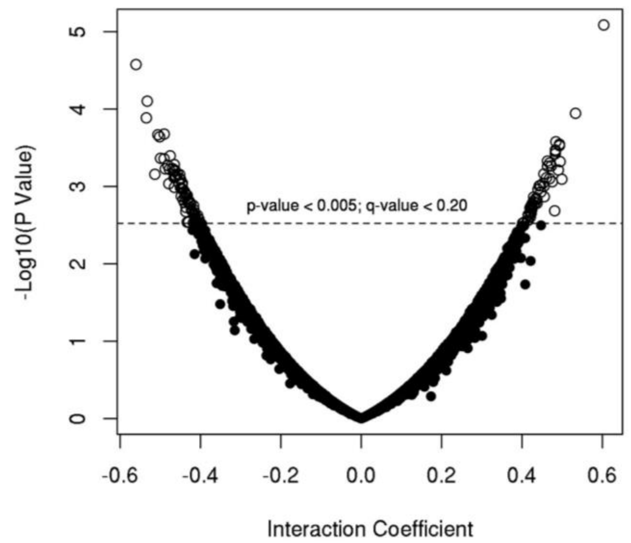

Figure S2. Significant findings from interaction model 1. The volcano plot visualizes the influence of $CD4^{+}$ T cell Day 28/Day 0 gene expression changes (per gene) on influenza A/H3N2 HAI titer (Day 28 - Day 0) by vaccine type (gene by vaccine interaction). The vertical axis represents -log10 of the p-value for the interaction term. The horizontal axis represents the interaction coefficient from interaction model 1. The open circles indicate the significant genes that have a q-value < 0.2 and p-value < 0.005 (n=128), and the significance threshold is represented by a dashed line.

Table S1. Influence of vaccine type and CD4⁺ T cell gene expression change on Ab response to influenza vaccination (Day 28-Day 0 HAI titer change). Full list of significant genes from interaction model 1. Top findings are summarized in Table 2.

Gene symbol	Entrezgene description	Estimate	p-value	q-value
SLC2A11	solute carrier family 2 member 11	0.6034	0.0000	0.0701
RP11-779O18.2	family with sequence similarity 58, member A (FAM58A) pseudogene	-0.5604	0.0000	0.1137
SLC38A5	solute carrier family 38 member 5	-0.5324	0.0001	0.1575
WDR35	WD repeat domain 35	0.5332	0.0001	0.1575
APOBR	apolipoprotein B receptor	-0.5347	0.0001	0.1575
CC2D1A	coiled-coil and C2 domain containing 1A	-0.4902	0.0002	0.1575
MYO9B	myosin IXB	-0.5059	0.0002	0.1575
PRKD2	protein kinase D2	-0.5016	0.0002	0.1575
SLC25A53	solute carrier family 25 member 53	0.4843	0.0003	0.1575
TMEM38B	transmembrane protein 38B	0.4931	0.0003	0.1575
B4GALT4	beta-1,4-galactosyltransferase 4	0.4931	0.0003	0.1575
SLC23A3	solute carrier family 23 member 3	0.4830	0.0003	0.1575
GORASP1	golgi reassembly stacking protein 1	0.4830	0.0004	0.1575
ERLIN2	ER lipid raft associated 2	0.4828	0.0004	0.1575
PITPNM1	phosphatidylinositol transfer protein membrane associated 1	-0.4753	0.0004	0.1575
MAP4	microtubule associated protein 4	-0.4993	0.0004	0.1575
MVB12B	multivesicular body subunit 12B	-0.4904	0.0004	0.1575
PKIG	cAMP-dependent protein kinase inhibitor gamma	0.4634	0.0005	0.1575
POP5	POP5 homolog, ribonuclease P/MRP subunit	0.4947	0.0005	0.1575
C17orf51	long intergenic non-protein coding RNA 2693	0.4690	0.0005	0.1575
WHSC1	nuclear receptor binding SET domain protein 2	-0.4651	0.0005	0.1575
PLEKHG5	pleckstrin homology and RhoGEF domain containing G5	-0.4809	0.0005	0.1575
PSMB5	proteasome 20S subunit beta 5	0.4728	0.0005	0.1575
SFXN2	sideroflexin 2	0.4729	0.0005	0.1575
CYP2U1	cytochrome P450 family 2 subfamily U member 1	0.4635	0.0005	0.1575
CHPF2	chondroitin polymerizing factor 2	-0.4694	0.0006	0.1575
RNASEK	ribonuclease K	-0.4876	0.0006	0.1575
TSC2	TSC complex subunit 2	-0.4772	0.0006	0.1575
TMEM14B	transmembrane protein 14B	0.4904	0.0006	0.1575
SF3A2	splicing factor 3a subunit 2	-0.4634	0.0006	0.1575
REXO1	RNA exonuclease 1 homolog	-0.4668	0.0006	0.1575
PGAP2	post-GPI attachment to proteins 2	0.4546	0.0007	0.1575
NUP214	nucleoporin 214	-0.4568	0.0007	0.1575
RHOG	ras homolog family member G	-0.5134	0.0007	0.1575
SSBP4	single stranded DNA binding protein 4	-0.4517	0.0007	0.1575
KMT2B	lysine methyltransferase 2B	-0.4636	0.0007	0.1575
SLC12A7	solute carrier family 12 member 7	-0.4611	0.0007	0.1575
PYGB	glycogen phosphorylase B	-0.4628	0.0008	0.1575
GTF3C1	general transcription factor IIIC subunit 1	-0.4656	0.0008	0.1575
PTPN20A	protein tyrosine phosphatase non-receptor type 20	0.4607	0.0008	0.1575
PREX1	phosphatidylinositol-3,4,5-trisphosphate dependent Rac exchange factor 1	-0.4518	0.0008	0.1575
NUDT3	nudix hydrolase 3	0.4687	0.0008	0.1575
CAMK4	calcium/calmodulin dependent protein kinase IV	0.4991	0.0008	0.1575
RBM42	RNA binding motif protein 42	-0.4494	0.0008	0.1575
TTC8	tetratricopeptide repeat domain 8	0.4753	0.0009	0.1598
FAM193A	family with sequence similarity 193 member A	-0.4450	0.0009	0.1598
NGRN	neugrin, neurite outgrowth associated	0.4526	0.0009	0.1598
ZBTB39	zinc finger and BTB domain containing 39	-0.4529	0.0009	0.1598
KDM4A	lysine demethylase 4A	-0.4787	0.0009	0.1598
PIAS3	protein inhibitor of activated STAT 3	-0.4458	0.0009	0.1598

NMNAT3	nicotinamide nucleotide adenylyltransferase 3	0.4461	0.0010	0.1598
RABL2A	RAB, member of RAS oncogene family like 2A	0.4427	0.0010	0.1598
VSIG10	V-set and immunoglobulin domain containing 10	0.4550	0.0010	0.1598
SPTAN1	spectrin alpha, non-erythrocytic 1	-0.4648	0.0010	0.1611
H1FX	H1.10 linker histone	-0.4496	0.0010	0.1611
LSR	lipolysis stimulated lipoprotein receptor	-0.4466	0.0011	0.1611
PRR12	proline rich 12	-0.4434	0.0011	0.1611
ZNF235	zinc finger protein 235	0.4398	0.0011	0.1611
TRAF7	TNF receptor associated factor 7	-0.4395	0.0012	0.1684
JADE1	jade family PHD finger 1	0.4468	0.0012	0.1684
BBS5	Bardet-Biedl syndrome 5	-0.4373	0.0013	0.1755
EGR1	early growth response 1	-0.4354	0.0013	0.1755
ZBTB49	zinc finger and BTB domain containing 49	-0.4329	0.0013	0.1755
C19orf47	chromosome 19 open reading frame 47	-0.4477	0.0013	0.1755
CENPI	centromere protein I	0.4553	0.0014	0.1755
SART1	spliceosome associated factor 1, recruiter of U4/U6.U5 tri-snRNP	-0.4390	0.0014	0.1755
FAM220A	family with sequence similarity 220 member A	0.4290	0.0014	0.1755
QDPR	quinoid dihydropteridine reductase	0.4353	0.0014	0.1776
CIC	capicua transcriptional repressor	-0.4319	0.0015	0.1780
ZNRD1	RNA polymerase I subunit H	0.4369	0.0015	0.1780
ZNF687	zinc finger protein 687	-0.4432	0.0015	0.1780
SP2	Sp2 transcription factor	-0.4283	0.0015	0.1783
OPA3	outer mitochondrial membrane lipid metabolism regulator OPA3	0.4282	0.0016	0.1783
AP3D1	adaptor related protein complex 3 subunit delta 1	-0.4326	0.0016	0.1783
DOPEY2	DOP1 leucine zipper like protein B	-0.4397	0.0016	0.1783
RABL2B	RAB, member of RAS oncogene family like 2B	0.4334	0.0016	0.1783
RPL24P4	RPL24 pseudogene 4	0.4373	0.0016	0.1783
NRROS	negative regulator of reactive oxygen species	-0.4277	0.0017	0.1783
ZNF319	zinc finger protein 319	-0.4238	0.0017	0.1783
COX6B1	cytochrome c oxidase subunit 6B1	0.4254	0.0017	0.1783
RP11-697E2.6	novel protein	0.4293	0.0017	0.1830
FAM161B	FAM161 centrosomal protein B	0.4249	0.0018	0.1830
NAA60	N-alpha-acetyltransferase 60, NatF catalytic subunit	-0.4171	0.0018	0.1830
AARSD1	alanyl-tRNA synthetase domain containing 1	0.4222	0.0018	0.1830
GATA3	GATA binding protein 3	-0.4358	0.0019	0.1861
MEST	mesoderm specific transcript	0.4199	0.0019	0.1861
BRD7P2	bromodomain containing 7 pseudogene 2	0.4243	0.0019	0.1861
RP4-592A1.2	adenylate kinase 2 (AK2) pseudogene	0.4200	0.0019	0.1861
BEX2	brain expressed X-linked 2	0.4245	0.0020	0.1887
IER2	immediate early response 2	-0.4159	0.0020	0.1887
BRPF1	bromodomain and PHD finger containing 1	-0.4225	0.0020	0.1887
CORO7-PAM16	CORO7-PAM16 readthrough	-0.4220	0.0020	0.1887
HMGN1	high mobility group nucleosome binding domain 1	0.4812	0.0021	0.1887
PLEKHB2	pleckstrin homology domain containing B2	0.4211	0.0021	0.1887
RAB43P1	RAB43 pseudogene 1	-0.4201	0.0021	0.1887
ZZEF1	zinc finger ZZ-type and EF-hand domain containing 1	-0.4364	0.0021	0.1890
C9orf3	aminopeptidase O (putative)	0.4204	0.0022	0.1928
ARMC10	armadillo repeat containing 10	0.4228	0.0022	0.1938
ZC3H3	zinc finger CCCH-type containing 3	-0.4160	0.0022	0.1938
SMURF1	SMAD specific E3 ubiquitin protein ligase 1	-0.4155	0.0023	0.1963
CDS2	CDP-diacylglycerol synthase 2	0.4152	0.0023	0.1967
ZNF592	zinc finger protein 592	-0.4072	0.0024	0.1967
TPT1P6	tumor protein, translationally-controlled 1 pseudogene 6	-0.4120	0.0024	0.1967
IL36A	interleukin 36 alpha	0.4159	0.0024	0.1967

MAP3K13	mitogen-activated protein kinase kinase kinase 13	0.4208	0.0025	0.1995
MRPL11	mitochondrial ribosomal protein L11	0.4204	0.0025	0.1995
CDC42EP2	CDC42 effector protein 2	-0.4123	0.0025	0.1995
TECRP1	trans-2,3-enoyl-CoA reductase pseudogene 1	-0.4138	0.0026	0.1995
FURIN	furin, paired basic amino acid cleaving enzyme	-0.4038	0.0026	0.1995
KIF13B	kinesin family member 13B	-0.4115	0.0026	0.1995
OSBPL7	oxysterol binding protein like 7	-0.4132	0.0027	0.1995
BCKDHA	branched chain keto acid dehydrogenase E1 subunit alpha	-0.4117	0.0027	0.1995
NEO1	neogenin 1	-0.4132	0.0027	0.1995
JUNB	JunB proto-oncogene, AP-1 transcription factor subunit	-0.4118	0.0027	0.1995
AP2A2	adaptor related protein complex 2 subunit alpha 2	-0.4109	0.0027	0.1995
ZNF444	zinc finger protein 444	0.4156	0.0027	0.1995
ACAP3	ArfGAP with coiled-coil, ankyrin repeat and PH domains 3	-0.4107	0.0027	0.1995
ZNF585A	zinc finger protein 585A	0.4132	0.0028	0.1995
SIPA1L1	signal induced proliferation associated 1 like 1	-0.4100	0.0028	0.1995
SOGA3	SOGA family member 3	-0.4294	0.0029	0.1995
KIAA0247	sushi domain containing 6	-0.4329	0.0029	0.1995
MRPS14	mitochondrial ribosomal protein S14	0.4054	0.0029	0.1995
WDR44	WD repeat domain 44	-0.4115	0.0029	0.1995
DHX30	DExH-box helicase 30	-0.4007	0.0029	0.1995
TLN1	talin 1	-0.4177	0.0030	0.1995
ZNF473	zinc finger protein 473	0.4046	0.0030	0.1995
ZYX	zyxin	-0.4139	0.0030	0.1995
SLC25A4	solute carrier family 25 member 4	0.4016	0.0030	0.1996

Table S2. Enriched biological pathways/processes involved in the immune outcome by vaccine type (from interaction model 2 [Day 0 gene expression effect] and from interaction model 3 [Day 28 gene expression effect]).

KEGG Pathway*	Set size	P-value	FDR***
**From the interaction model 2**			
Oxidative phosphorylation	62	1.056E-06	0.0004
Citrate cycle (TCA cycle)	15	0.0002	0.0243
Signaling pathways regulating pluripotency of stem cells	60	0.0007	0.0495
Prion disease	123	0.0007	0.0495
**From the interaction model 3**			
**Antigen processing and presentation****	42	7.50781E-07	0.0001
Osteoclast differentiation	76	4.70354E-06	0.0002
**Hematopoietic cell lineage****	62	5.75942E-06	0.0002
Kaposi sarcoma-associated herpesvirus infection	101	0.0001	0.0034
Influenza A	91	0.0001	0.0034
B cell receptor signaling pathway	49	0.0001	0.0034
**Th1 and Th2 cell differentiation****	50	0.0001	0.0034
**Th17 cell differentiation****	59	0.0002	0.0036
Viral myocarditis	37	0.0003	0.0058
**NOD-like receptor signaling pathway****	95	0.0004	0.0064
PD-L1 expression and PD-1 checkpoint pathway in cancer	45	0.0004	0.0066
Phagosome	81	0.0005	0.0069
**Cell adhesion molecules****	73	0.0005	0.0070
Protein digestion and absorption	39	0.0005	0.0070
C-type lectin receptor signaling pathway	56	0.0007	0.0087
Epstein-Barr virus infection	114	0.0008	0.0096
**NF-kappa B signaling pathway****	65	0.0010	0.0113
Neutrophil extracellular trap formation	87	0.0012	0.0120
**Glycolysis / Gluconeogenesis****	27	0.0027	0.0261
Fc gamma R-mediated phagocytosis	59	0.0033	0.0296
Citrate cycle (TCA cycle)	15	0.0036	0.0313
Glycosaminoglycan biosynthesis - keratan sulfate	8	0.0049	0.0416
Viral carcinogenesis	110	0.0058	0.0473
**Apoptosis****	23	0.0062	0.0498

*From the enriched pathways list we removed pathways associated with disease, that arouse from combinations of other pathways

**Pathways related to T cell differentiation, activation and function are bolded.

***FDRs were calculated using Benjamini-Hochberg method (Methods).
